# Unraveling the Nectar Secretion Pathway and Floral-Specific Expression of *SWEET* and *CWIV* Genes in Five Dandelion Species Through RNA Sequencing

**DOI:** 10.3390/plants14111718

**Published:** 2025-06-05

**Authors:** Sivagami-Jean Claude, Sunmi Park, Seong-Jun Park, SeonJoo Park

**Affiliations:** 1 Department of Life Sciences, Gachon University, 1342, Seongnamdaero, Seongnam-si 13120, Republic of Korea; sivajeankor@gmail.com; 2 Department of Life Sciences, Yeungnam University, Gyeongsan 38541, Republic of Korea

**Keywords:** floral nectar, *SWEET*, *CWIN*, dandelions, *Taraxacum*, transcriptome

## Abstract

*Taraxacum*, a genus in the Asteraceae family, is widely distributed across temperate regions and plays a vital ecological role by providing nectar and pollen to pollinators during the early flowering season. Floral nectar is a key reward that plants use to attract pollinators, and its production is tightly regulated by genes such as *SWEET* sugar transporters and *CELL WALL INVERTASE* (*CWIN*), which govern sugar efflux and hydrolysis. Despite their ecological importance, the molecular mechanisms underlying nectar secretion in *Taraxacum* remain poorly understood. In this study, we performed RNA sequencing of flower tissues from five *Taraxacum* species—*T. coreanum*, *T. monogolicum*, *T. ohwianum*, *T. hallaisanense*, and *T. officinale*—to investigate the expression of nectar-related genes. De novo transcriptome assembly revealed that *T. coreanum* had the highest unigene count (74,689), followed by *T. monogolicum* (69,234), *T. ohwianum* (64,296), *T. hallaisanense* (59,599), and *T. officinale* (58,924). Functional annotation and phylogenetic analyses identified 17 putative *SWEET* and 18 *CWIN* genes across the five species. Differential gene expression analysis highlighted *tarSWEET9* and *tarCWIN4* as consistently up-regulated during the flowering stage. Quantitative PCR in *T. officinale* further validated that *tarSWEET9*, *tarCWIN4*, *tarCWIN6*, and *tarSPAS2* show significant expression during floral development but are down-regulated after pollination. These genes are likely central to the regulation of nectar secretion in response to pollination cues. Our findings suggest that *T. officinale* may have evolved to have an efficient, pollinator-responsive nectar secretion system, contributing to its global adaptability. This study sheds light on how pollinator interactions influence gene expression patterns and may drive evolutionary divergence among *Taraxacum* species.

## 1. Introduction

Floral nectar (FN) and pollen have evolved in plants as rewards to attract pollinators [1,2,3,4,5]. Pollen, the male gametophyte of land plants, is produced in cones in gymnosperms and in anthers within the stamens in angiosperms. For successful reproduction in flowers, pollen is initially dispersed by insects or wind [6]. Nectar contains sugars and amino acids (AAs) and is associated with specific responses to pollinators [7,8]. It is primarily composed of a sucrose-rich aqueous solution, with lesser amounts of glucose, fructose, amino acids, and enzymes. These components vary among species and are collectively referred to as a hexose solution [9,10]. During nectary development and nectar secretion, starch grains accumulate and subsequently break down in most plants. To establish a mutually beneficial relationship with insects, plants offer floral nectar (FN) through nectaries located at the base of the floral thalamus, between the androecium and gynoecium [11,12]. Most angiosperm nectaries consist of three primary components: the epidermis, specific parenchyma cells, and vascular bundles [13,14].

Nectaries have been analyzed across various angiosperm species, exhibiting structural diversity within the epidermis, hypodermis, and trichomes. Studies indicate that 94% of angiosperms are animal-pollinated [15,16,17]. Nectar synthesis primarily occurs through sucrose biosynthesis and is facilitated by specific transporters, which serve as candidate genes for nectar production in *Arabidopsis* [18]. Pollinator visits often increase nectar flow; however, nectar production ceases post-pollination, and any remaining nectar is reabsorbed [19]. Nectar production is regulated by two distinct secretion processes: eccrine and granulocrine secretion, which vary among plant species [20,21,22]. The *SWEET* gene family encodes sugar transporters responsible for regulating the movement of sugars across the cytoplasm, particularly in conditions of low sugar demand [23,24]. These *SWEET* transporters can facilitate the absorption and release of sugar molecules within cells without relying on energy-dependent uniporters [25]. Nectar diffusion occurs through three different mechanisms: sugar movement from the apoplasm, cytoplasmic sugar transport via symplasms, and diffusion from the phloem [21,26,27]. The morphology, production, and biological significance of floral nectaries have been studied across multiple angiosperm lineages, including *Arabidopsis*, *Borago*, *Euonymus*, *Lamprocapnos*, *Pisum*, *Parnassia*, *Opuntia*, and *Cucurbita* species [21,22,28,29].

Genome-wide analyses have identified *SWEET* and *CWIN* genes in a wide range of land plants, including representative dicotyledonous species such as *Arabidopsis thaliana* [30], *Vitis vinifera* [31], *Solanum lycopersicum* [32], and *Glycine max* [33], and monocotyledonous species like *Oryza sativa* [34] and *Triticum aestivum* [35]. These genes play key roles in the nectar secretion pathway by regulating sugar transport and hydrolysis. Notably, *SWEET*9 and *CWINV*4 have been shown to be essential for nectar production in *Arabidopsis*, where their knockout results in significantly reduced nectar secretion [30]. Overexpression of *SWEET* genes in *Oryza* and *Solanum* has also been linked to changes in sugar distribution and floral development [32,34]. Similarly, silencing of *CWIN* genes in tomato disrupted sucrose metabolism and reproductive development [36,37]. Together, these findings highlight the conserved functional roles of *SWEET* and *CWIN* genes in floral organ physiology and nectar production.

*Taraxacum* (dandelion) is a perennial herb belonging to the family Asteraceae. It is an apomictic species that reproduces asexually by dispersing seeds through wind. Dandelions are widely distributed throughout the Northern Hemisphere, with over 2500 species reported [38,39,40,41,42]. *Taraxacum* is a taxonomically complex genus comprising both diploid and polyploid species, with polyploidy being especially prevalent. Diploid *Taraxacum* species typically reproduce sexually, while most polyploids reproduce through apomixis (asexual seed formation). The ploidy levels can range from diploid (2n = 2x = 16) to triploid and higher, with triploids (3x = 24) being the most common among apomictic dandelions [43,44]. Dandelions, also referred to as monofloral honey plants, serve as an essential food source for many insects due to their early flowering and abundant supply of pollen and nectar [45,46]. Notably, nectar production in dandelions is highly dependent on the flowering stage. *Taraxacum* species have been shown to exhibit significant variation in nectar quantity and sugar content under natural conditions, highlighting their ecological relevance and supporting the need to investigate the genetic basis of nectar secretion through transcriptomic approaches [47,48]. In this study, we selected flowers from five *Taraxacum* species to investigate genes involved in nectar biosynthesis and their secretion mechanisms using single-RNA sequencing. Although *Taraxacum* species are considered prolific nectar producers [39], the relationship between nectary modifications and variations in nectar secretion among species has not yet been explored at the genetic level. Furthermore, the identification and characterization of *SWEET* and *CWIN* genes at a transcriptome-wide level remain unexplored in the Asteraceae family. This study provides fundamental insights into the potential functional roles of *SWEET* and *CWIN* gene discoveries in *Taraxacum*. We identified and characterized 16 *tarSWEET* and 18 *tarCWIN* genes, analyzing their differential expression patterns across five *Taraxacum* species. High-throughput RNA sequencing was employed to characterize genes associated with nectar secretion and related metabolic pathways. Additionally, quantitative RT-PCR was performed to examine the expression of nectar-related sugar metabolism and male-sterility genes in *Taraxacum* flowers at distinct stages of development. Understanding the nectar secretion pathway in *Taraxacum* may provide insights into how these species have evolved to utilize both apomixis and cross-pollination strategies while attracting pollinators through nectar production.

This study offers a comprehensive analysis of differential gene expression patterns associated with the nectar secretion pathway, shedding light on the functional roles of *tarSWEET* and *tarCWIN* gene families. Our findings contribute valuable information for further exploration of *SWEET* and *CWIN* gene functions in *Taraxacum*. Moreover, this study provides novel insights into the nectar secretion mechanisms, specifically through the merocrine or eccrine model, in five different *Taraxacum* species using next-generation sequencing.

## 2. Results

### 2.1. Assembly and Functional Annotation

The de novo assembly of individual *Taraxacum* species yielded the highest number of transcripts in *T. coreanum* (74,689 unigenes), while *T. officinale* exhibited the lowest count (Table 1). The combined de novo assembly of all five species resulted in a total of 197,473 unigenes from *Taraxacum* flower sequencing. The quality of transcriptome assembly was assessed using BUSCO, with the combined assembly achieving a completeness score of 91.6%. Among individual species, *T. mongolicum* showed the highest completeness (93.6%), followed by *T. officinale* (93.5%), *T. ohwianum* (93.0%), *T. coreanum* (92.6%), and *T. hallaisanense* (82.2%) (Supplementary Figure S2). Functional annotation using BLAST against the TAIR database revealed unigene assignments of 44.34% (*T. coreanum*), 33.07% (*T. mongolicum*), 26.78% (*T. officinale*), 30.02% (*T. hallaisanense*), and 25.55% (*T. ohwianum*). Similarly, alignment against the Swiss-Prot database identified 47.36% of unigenes in *T. coreanum*, 38.90% in *T. ohwianum*, 37.59% in *T. mongolicum*, 34.59% in *T. hallaisanense*, and 29.78% in *T. officinale*. KEGG pathway annotation further categorized unigenes into functional pathways, with distributions consistent with TAIR-based annotation (Table 1).

### 2.2. Gene Ontology (GO) Terms Associated with Sucrose Biosynthesis in Taraxacum

GO analysis of functionally annotated transcripts related to sucrose metabolism was performed using the TAIR database (Figure S3A, Supplementary Table S1). A total of 8238 transcripts were associated with biological processes and cellular components. Of these, 2408 genes were annotated to localization processes, including secretion, nectar secretion, and transport (Supplementary Figure S3A). Carbohydrate metabolic processes accounted for 308 unigenes, including disaccharide metabolism (70), oligosaccharide metabolism (80), and sucrose metabolism (46).

In the category of organic transport, 1001 unigenes were identified, with 63 related to carbohydrate transport, 20 to disaccharide transport, 21 to oligosaccharide transport, and 20 to sucrose transport in *Taraxacum* flowers. Additionally, 1643 unigenes were assigned to responses to organic substances, including responses to carbohydrates (153), cellular responses to oxygen-containing compounds (518), monosaccharide stimuli (72), hexose (68), and hexose-mediated signaling (7).

For monosaccharide metabolism, 128 unigenes were identified and categorized as hexose metabolism (87), fructose metabolism (13), and fructose response (15). Further GO terms included metabolic starch processes (64 unigenes), glucose responses (63), and glucose metabolism (42), which were classified under biological processes. Additionally, genes associated with reproductive structure development included 738 unigenes involved in seed development and 765 in fruit development (Supplementary Figure S3A).

Molecular function analysis revealed that 40.38% of genes were associated with carbohydrate transmembrane processes and transport activity. Sugar transmembrane transport activity was specifically represented by 28.85% of the genes (Supplementary Figure S3B). Cellular component analysis indicated that 30.57% of genes were associated with cellular and anatomical entities, 21.40% were classified under membrane-related functions, and 28.85% were linked to intrinsic and integral membrane components (Supplementary Figure S3C).

### 2.3. KEGG Annotation

KEGG pathway analysis was performed on the assembled unigenes of five *Taraxacum* species (Table 1, Supplementary Figure S4). A total of 397,344 transcripts from the combined assembly were submitted for KEGG annotation, of which 109,162 (27.55%) were assigned to various metabolic pathways. Among the annotated pathways, the highest proportion of unigenes were associated with protein families involved in protein families genetic information processing (20.24%), followed by general genetic information processing (16.37%), signaling and cellular processes (8.46%), carbohydrate metabolism (8.32%), secondary metabolism (6.88%), amino acid metabolism (2.87%), and terpenoid and polyketide metabolism (1.64%) (Supplementary Figure S4A). Additionally, genes involved in nectar secretion pathways were identified, including those associated with amino acid biosynthesis (111 unigenes), starch and sucrose metabolism (40), amino sugar and nucleotide sugar metabolism (54), fatty acid metabolism (46), and fructose and mannose metabolism (22) (Supplementary Figure S4B).

### 2.4. SWEET/CWIN/MS Genes and Their Phylogeny

**Figure 1:**
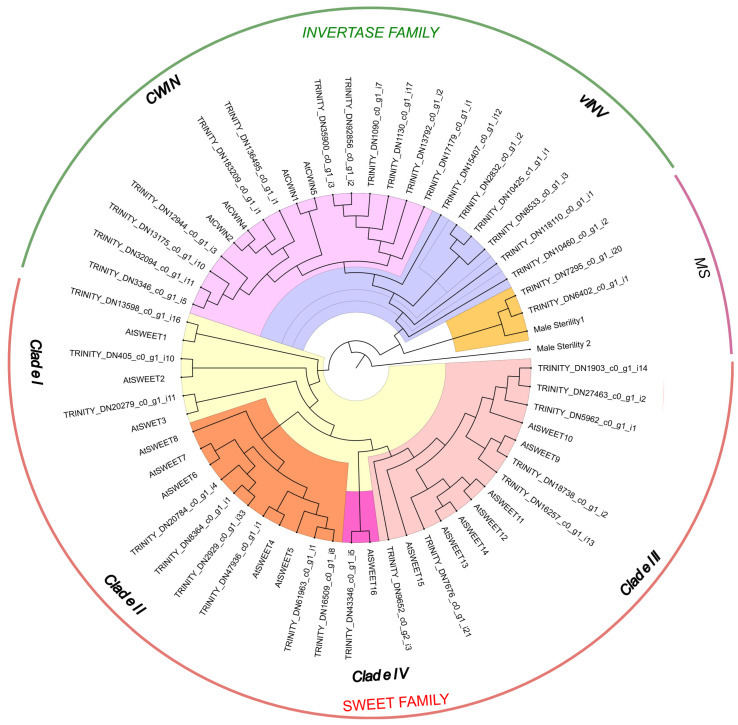
Phylogenetic analysis of *SWEET*, *Invertase* (INV), and *Male Sterility* (MS) genes in the *Taraxacum* genus and *Arabidopsis.* A total of 59 protein sequences (TAIR + *Taraxacum* unigenes) were used to construct the maximum likelihood (ML) tree with 1000 bootstrap replicates in IQ-TREE. The tree was midpoint-rooted using FigTree. *Taraxacum* phylogroups are color-coded to represent gene categories: *MS* (*Male Sterility*), *CWIN* (*Cell Wall Invertase*), *vINV* (*Vacuolar Invertase*), and *SWEET*.

A phylogenetic analysis was conducted using *SWEET* and *CWIN* genes identified in *Taraxacum* flowers, along with 16 *AtSWEET* and 4 *AtCWIN* genes from *Arabidopsis thaliana* (Figure 1).

SWEET proteins exhibited a highly conserved amino acid length ranging from 171 to 227 residues, with two characteristic PQ (Pro and Gln) loop repeat domains. The identified *TarSWEET* genes were classified into four clades:

- Clade I (*SWEET*1–3) and Clade II (*SWEET*4–8) were associated with glucose transport.
- Clade III (*SWEET*9–15) participated in sucrose transport.
- Clade IV (*SWEET*16) was linked to fructose transport.

**Figure 2:**
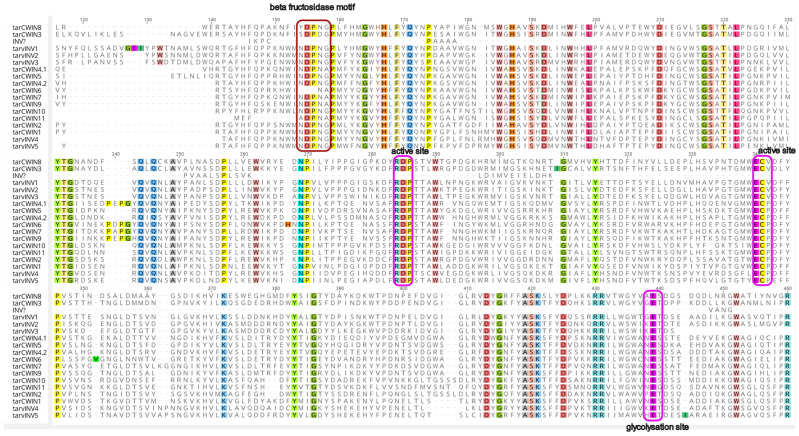
MUSCLE alignment of 18 unigenes of *Taraxacum invertase* family members. *INV*-*CW* protein active sites, beta fructosidase motifs, glycosylation sites of *CWIN* (NES), and vacuolar *INV* (*vINV*) (GET) sites were identified.

A total of 17 SWEET genes were discovered in *Taraxacum*, corresponding to similar groupings found in Arabidopsis. Additionally, two pseudogenes with incomplete Gln motifs were identified but excluded from phylogenetic analysis (Figure 1). Using four *AtCWIN* sequences as queries, 18 *INV* genes were identified in *Taraxacum* (Figure 1). *CWIN* proteins exhibited a conserved amino acid length of 415 to 665 bp. Among the 18 *Taraxacum CWIN* genes, 2 copies were closely related to *AtCWIN*, and the remaining genes were distributed into three major clades, with 14 belonging to a distinct *Taraxacum* flower-specific *CWIN* cluster. Furthermore, two *MS* genes were identified, but only two homologs to *Arabidopsis* were confirmed. To re-examine *CWIN* and other *INVERTASE* genes in *Taraxacum*, sequence alignments were performed to identify conserved motifs of *CWIN* and vacuolar invertases (*vINVs*) (Figure 2). A total of twelve genes were classified as *CWIN,* and six were classified as *vINV*. All *Taraxacum* flower *SWEET*, *CWIN*, and *MS* gene sequences were submitted to NCBI GenBank under accession numbers ON351066–ON351241.

### 2.5. DEG Analysis of SWEET/CWIN/MS Genes in Taraxacum Flowers

**Figure 3:**
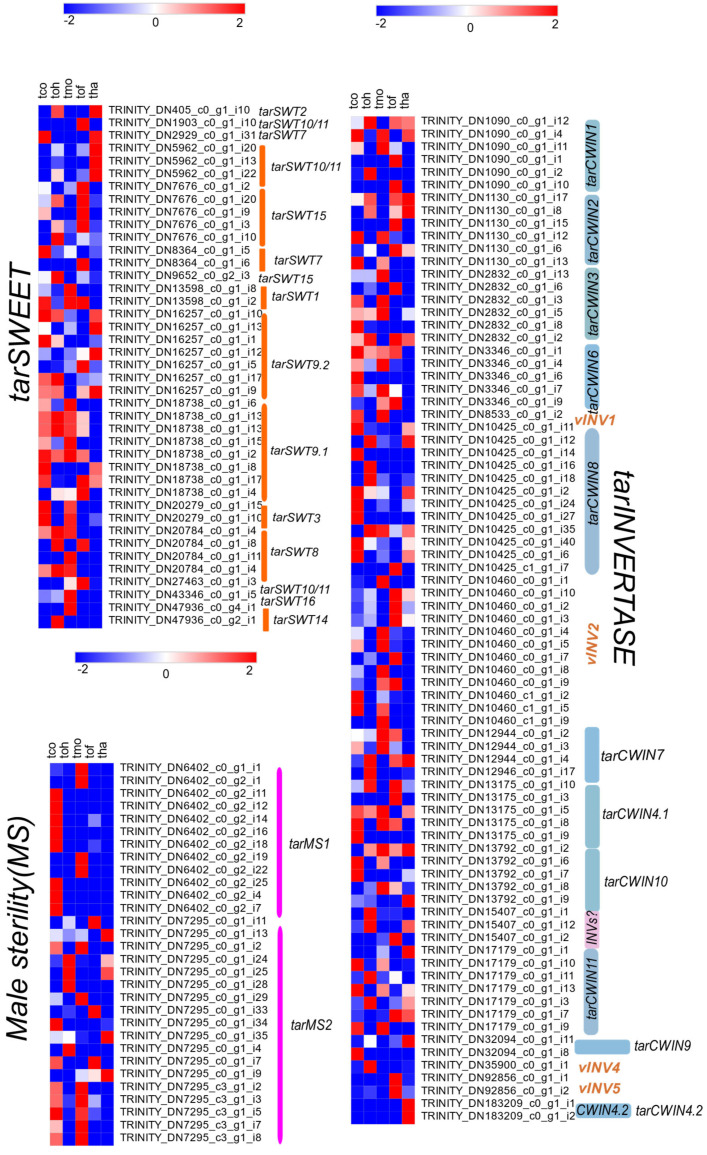
Heatmaps of *SWEET*/*CWIN*/*MS* unigenes were produced from DEG analysis. tha: *T. hallaisanense*; tmo: *T. mongolicum*; toh: *T. ohwianum*; tof: *T*. *officinale*; tco: *T. coreanum*.

We identified 130,294 differentially expressed unigenes (DEGs) at a log2 fold change threshold across five *Taraxacum* species, based on a complete set of 655,076 transcripts. Relative expression values were obtained using the NOISeq and Trinity-RSEM pipeline (Supplementary Figure S3). The DEG correlation matrix indicated that *T. coreanum* and *T. mongolicum* are closely related, while *T. officinale* forms a sister clade with *T. ohwianum* and *T. hallaisanense* (Supplementary Figure S4). The highest number of up-regulated genes was observed in *T. mongolicum* (34,405), whereas *T. officinale* exhibited the lowest number (25,644). Similarly, *T. officinale* had the highest number of down-regulated genes, while *T. hallaisanense* had the lowest (Supplementary Figure S3A). A total of 16 *SWEET* genes, represented by 42 transcripts, were identified. Among them, seven were up-regulated, and nine were down-regulated in *T. coreanum*, *T. ohwianum*, and *T. mongolicum*. In *T. officinale*, nine unigenes were up-regulated, and seven were down-regulated. Additionally, two *SWEET* genes were uniquely present in *T. officinale*. *T. hallaisanense* exhibited the lowest number of up-regulated (six) and down-regulated (ten) genes (Figure 3, Supplementary Table S3).

The *CWIN* expression heatmap revealed that 17 genes were shared among *Taraxacum* flowers. Of these, eleven genes were up-regulated, and six were down-regulated in *T. coreanum*, *T. ohwianum*, *T. mongolicum*, and *T. hallaisanense*. In *T. officinale*, thirteen unigenes were up-regulated, while four were down-regulated. Furthermore, apart from the seventeen DEGs, one additional *CWIN* gene was detected in *T. officinale* (Figure 3, Supplementary Table S4). Interestingly, both copies of *MS* genes were up-regulated in *T. coreanum* and *T. mongolicum* only. In contrast, in *T. hallaisanense*, *T. ohwianum*, and *T. officinale*, only one copy of *MS* was up-regulated, and the other was down-regulated (Supplementary Table S5).

### 2.6. Analysis of Nectar Secretion Pathway and Associated Gene Expressions in Taraxacum Flowers

**Figure 4:**
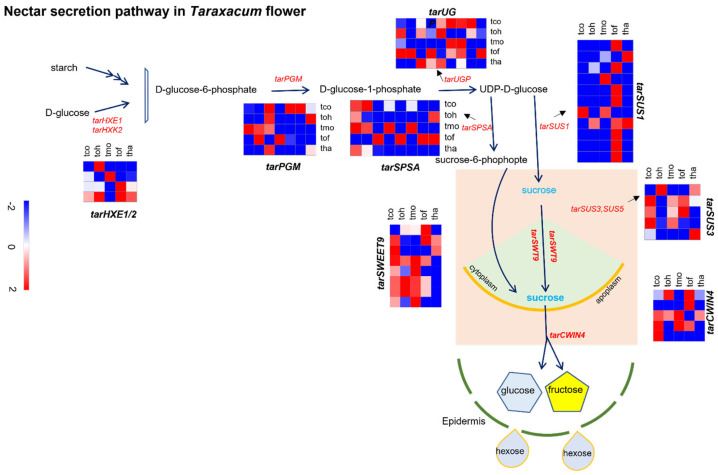
Differentially expressed genes (DEGs) were identified in the nectar secretion pathway as well as genes involved in sucrose production, transport, and hydrolysis in *Taraxacum*. The following genes were examined: *HXE*, *hexokinase* 1/2; *PGM2*, *phosphoglucomutase 2*; *UGP*, *UTP-glucose-1-phosphatase*; *SPSA*2, *sucrose phosphate synthase* 2; *SUS*1, *sucrose transport*1,3,5; *CWIN*, *cell wall invertase* 4. The sucrose metabolic pathway was constructed using the Plant Metabolic Network (PMN). Most genes associated with nectar secretion were up-regulated in *Taraxacum*. Detailed DEG analysis and transcripts of sucrose biosynthesis are provided in Supplementary Table S6. The species examined include tha: *T. hallaisanense*; tmo: *T. mongolicum*; toh: *T. ohwianum*; tof: *T*. *officinale*; and tco: *T. coreanum.*

Genes associated with hexose solution (nectar) secretion in *Taraxacum* flowers were analyzed through gene BLAST and DEG analysis (Figure 4). The nectar secretion pathway was identified in *Taraxacum* flowers, which involves the conversion of D-glucose into D-glucose-6-phosphate by five *Sucrose Synthase 1* (*tarSUS*1) genes that are responsible for converting UDP-D-glucose into sucrose. Two *tarSUS*1 unigenes were up-regulated, while three were down-regulated in *T. hallaisanense* and *T. mongolicum*. In *T. ohwianum* and *T. coreanum*, one unigene was up-regulated, while four were down-regulated. Notably, all five *tarSUS*1 unigenes were up-regulated in *T. officinale* but exhibited multiple isoform variations (Table S6). The two-way synthesis of sucrose involves the conversion of *UDP*-*D*-*glucose* into *Sucrose*-6-phosphate, which is catalyzed by *SPSA*2. Three unigenes were associated with *tarSPSA*2 in *Taraxacum* flowers. In *T. hallaisanense* and *T. coreanum*, one unigene was up-regulated, while two were down-regulated. In *T. ohwianum*, *T. mongolicum*, and *T. officinale*, two unigenes were up-regulated, and one was down-regulated (Figure 4). Comparative analysis with *Arabidopsis* revealed that both copies of *tarSWEET9* were up-regulated in all *Taraxacum* species except *T. mongolicum*. Additionally, 12 unigenes were identified as homologous to the *Arabidopsis class CWIN* genes, possessing conserved *INV-CWIN* catalytic motifs (*NES* and *GET*). Among these, four *CWIN* genes were up-regulated, and one was down-regulated in *T. officinale* and *T. hallaisanense*. Meanwhile, three *tarCWIN4* genes were up-regulated, and two were down-regulated in *T. ohwianum*, *T. coreanum*, and *T. mongolicum*, clustering within Clade III (Supplementary Table S4).

The *Taraxacum* nectar secretion pathway involved hexokinase 1 and 2 (*HXK1* and *HXK2*). Four transcript variants of *tarHXK1* and *tarHXK2* were identified. Two *tarHXK* genes were up-regulated in *T. ohwianum* and *T. officinale*, while one copy was up-regulated in *T. hallaisanense*, *T. coreanum*, and *T. mongolicum*. *Phosphoglucomutase* 2 (*tarPGM*2) catalyzes the conversion of *D-glucose-6-phosphate* to *D-glucose-1-phosphate*. Three copies of *tarPGM* were identified: two were up-regulated in *T. coreanum*, *T. ohwianum*, and *T. officinale*, while in *T. mongolicum* and *T. hallaisanense*, one was up-regulated, and two were down-regulated. The conversion of D-glucose-1-phosphate to UDP-D-glucose is catalyzed by *UTP-glucose-1-phosphatase* 2 (*tarUGP*2). This gene was found in five copies: two were up-regulated in *T. coreanum*, *T. ohwianum*, and *T. hallaisanense*, while *T. mongolicum* exhibited one up-regulated and four down-regulated copies. In *T. officinale*, three copies were up-regulated, and two were down-regulated, making it the species with the highest copy number of *tarUGP* genes (Figure 4, Supplementary Table S6).

### 2.7. Quantification of Genes Involved in the Nectar Secretion Pathway in T. officinale

**Figure 5:**
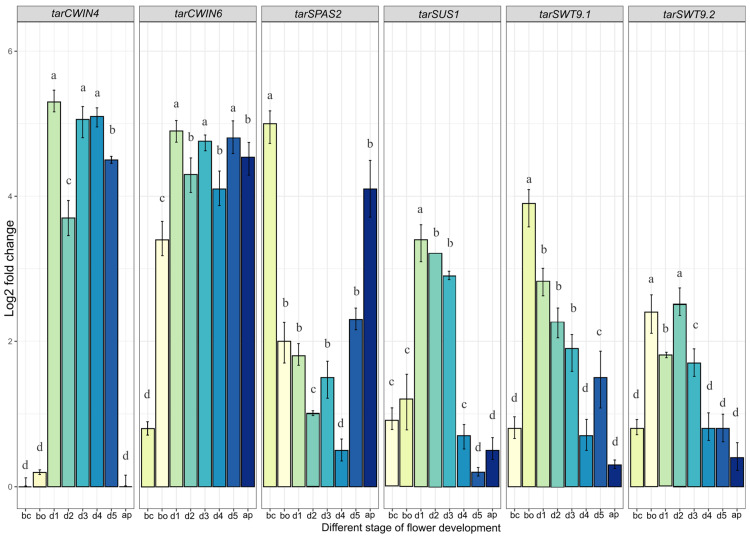
Quantification of nectar secretion pathway-related unigenes from day 1 to 5, with bud closed (bc) and bud opened (bo), and during after-pollination (ap), a distinct stage of *T*. *officinale*, was analyzed. The error bar indicates the mean ± SD. Significance was assessed using a one-way ANOVA, and non-overlapping letters (a–d) above the bars indicate groups that are statistically different (*p* < 0.05) according to Tukey’s HSD post hoc test. *SWT*: *SWEET*; *SPSA*2: *sucrose phosphate synthase* 2; *SUS*1: *sucrose transport* 1; *CWIN*: *cell wall invertase 4/6*.

To validate the involvement of key genes in the nectar secretion pathway, six nectar-associated genes were selected for quantitative analysis. Gene expressions were assessed across different flowering stages to elucidate the roles of these genes in nectar secretion during floral development. qPCR analysis revealed that *tarSPAS* and *tarSUS*1 exhibited differential expression patterns across flower developmental stages, suggesting that sucrose transport in *T. officinale* is primarily mediated by *tarSPAS*, rather than through sucrose conversion from UDP-glucose by *tarSUS*1 (Figure 5). Specifically, *tarSPAS* showed high expression levels before and after flower opening and on day 1, followed by moderate expression on day 5 and at the post-fertilization (after pollination) stage. This pattern suggests that *tarSPAS* plays a significant role in flower development, whereas *tarSUS*1 may have a more limited function. Further analysis of *tarSWEET*9.1 and *tarSWEET*9.2 revealed that *tarSWEET9.2* was more actively involved in sucrose transport than *tarSWEET*9.1 during flowering. Expression profiling of *tarCWIN6* demonstrated significant up-regulation from dawn through day 1 and sustained expression until day 5. In contrast, *tarCWIN4* was significantly expressed from flower opening (day 1 to day 5) until after pollination but showed minimal expression before flower opening (Figure 5).

## 3. Discussion

Nectar is a sugar-rich solution secreted by specific angiosperms to attract pollinators, facilitating pollination [14,49]. It has evolved primarily in female flowers and varies between male and female flowers in higher plants [50]. Additionally, nectar can deter certain species from visiting or engaging in other biological interactions [4]. It contains various components, including sugars, amino acids, alkaloids, glycosides, flavonoids, phenolics, vitamins, ions, proteins, and free fatty acids [8]. Both nectar and pollen have been extensively studied in the context of plant evolution, genetics, physiology, and ecology [3,51]. In this study, we performed RNA sequencing (RNA-Seq) on five *Taraxacum* species to analyze nectar secretion mechanism-related genes. While *Taraxacum* species primarily rely on cross-pollination for seed dispersal, they can also undergo self-pollination through triploid or tetraploid pollen when cross-pollination is unsuccessful [52,53].

The *SWEET* gene family, known for its role in sugar transport, as well as plant seed and pollen development, has been identified in over 27 plant species [54,55,56,57]. It plays a crucial role in plant growth, development, and stress responses [23,57,58]. A study on zucchini nectar and pollen visits revealed that a higher sucrose-to-hexose ratio in nectar attracted more pollinator visits [59]. Comparative genome-wide studies have identified *SWEET* genes in various plant species: 17 in *Arabidopsis* [23], 17 in *Vitis* [31], 21 in *Oryza* [34], 10 in *Averrhoa* [60], 27 in *Citrus* [61], 19 in *Hemerocallis* [62], 52 in *Eucalyptus* [63], 28 in *Camellia* [64], 13 in *Poa* [65], 20 in octoploid *Fragaria* X *ananassa* [66], 108 in *Triticum* [35], 22 in *Citrullus* [67], 16 in *Litchi* [68], 17–26 in *Cucumis* species [69,70], 60 in *Glycine* [33,71], 25 in *Medicago* [72], 36 in *Hevea* [73], 16 in *Brassica* [74], 68 in *Malus* [75], 25 in *Musa* [76] and 28 in *Solanum* [32]. Genome-wide studies in *Taraxacum kok-saghyz* identified 22 *SWEET* genes, with *SWEET1* and *SWEET12* specifically implicated in cytoplasmic functions within rubber-producing laticifers, suggesting a role in rubber biosynthesis [77]. In our study, we identified only the *tarSWEET1* gene, which was strongly up-regulated in all species except *T. mongolicum*, suggesting its potential role in latex synthesis across most *Taraxacum* species.

Phylogenetic analysis of *Arabidopsis SWEET* genes supports a four-clade division, which is also reflected in *Taraxacum*: Clade I (three genes), Clade II (six genes), Clade III (seven genes), and Clade IV (one gene) [23]. Clade III of the *SWEET* family, particularly *SWT9*, facilitates sucrose transport from the cytoplasm and functions as an efflux transporter [78]. *SWEET* genes also influence fruit development and ripening [79,80]. We identified 17 *SWEET* genes in *Taraxacum* and analyzed their expression through differential expression gene (DEG) analysis. Six unigenes belonged to Clade III, which is responsible for sucrose transport by phloem parenchymal cells [24,81]. DEG analysis revealed that *tarSWEET*10 genes were up-regulated in *T. hallaisanense* and *T. ohwianum*, indicating regional or species-specific bidirectional sucrose transport activity in leaves and shoot apices. In *T. officinale*, two unique *tarSWEET*10 and *tarSWEET*11 genes were up-regulated, suggesting their role in accelerated flowering and sugar transport [82]. *SWEET*10 has been identified as being expressed in the shoot apex during floral transition, suggesting its role in the transport of gibberellins and sucrose in potato [83]. In contrast, *SWEET*11 plays a critical role in seed filling and is essential for facilitating sugar efflux from the nucellar epidermis as well as the ovular vascular trace into the apoplast in rice [84,85].

Expression analysis of *SWEET* gene family Clade III and IV genes revealed that *tarSWEET15* was up-regulated exclusively in *T. officinale* and *T. ohwianum*, while it was down-regulated in other *Taraxacum* species. *SWEET15* is known to play a key role in two major steps of apoplasmic seed filling, as previously observed in rice and barley [84,85,86]. Similarly, both *tarSWEET*16/17 were regulated in *T. mongolicum* while being down-regulated in other species. In *Arabidopsis*, *SWEET15* and *SWEET16* are strongly induced during senescence and osmotic stress, suggesting that *tarSWEET15* and *tarSWEET16* play roles in stress response in *Taraxacum* flowers [87,88,89]. Whereas the *SWEET*15/17 gene functions as a vacuolar sugar facilitator and is primarily expressed in vascular parenchyma cells, the overexpression of *SWEET16* has been shown to alter sugar accumulation [88]. Under stress conditions, *SWEET16* plays roles in cold stress response, nitrogen starvation, enhanced germination under cold conditions, and improved salinity tolerance in *Arabidopsis* [90].

The sucrose-specific gene *SWEET*9, located in the cytoplasm, is activated in response to increased sugar concentration [78]. In our study, we identified two genes related to *tarSWEET9*, one of which was up-regulated in four *Taraxacum* species but down-regulated in *T. mongolicum*, suggesting that most of the *Taraxacum* species are capable of nectar secretion during pollination [24,78]. Quantification of two *tarSWEET*9 gene copies in *T. officinale* revealed that *tarSWEET*9 and other nectar secretion-related genes were highly expressed in closed and open buds [18]. This pattern aligns with a previous report on nectar secretion in *Cucurbita*, where peak nectar production occurred before and after the opening of the buds, followed by a decline approximately nine hours post-anthesis [91]. We observed that *tarSWEET9* expression remained significant in closed and open buds and was maintained at moderate levels from days 1 to 6 in *T. officinale*, with Clade III *SWEET* genes being highly expressed in *Taraxacum*. Interestingly, our quantification assay showed that *tarSWEET*9, *tarCWIN*4, and *tarSUS*1 expression levels declined after pollination, which indicates the previously accepted pattern of nectar secretion before and after pollination.

The enzyme *CWIN*4, localized in the cell wall, hydrolyzes sucrose into glucose and fructose within the apoplast. This process increases both intra- and extracellular sucrose concentrations, leading to the efflux of water and sugar molecules into the apoplast, forming nectar droplets through epidermal cells during stomatal opening. The glycosylation sites of the *CWIN* and *vINV* genes have been previously identified [92]. Additionally, our study identified six *vINV*-related genes, which may function as hexose transporters, facilitating the mobilization of stored vacuolar sucrose during nectar secretion [93]. These findings provide valuable insights into the differential gene expression patterns in *Taraxacum* species and their functional implications in sucrose metabolism and regulatory pathways.

In our analysis, we identified 12 copies of *CWIN* and 6 copies of *vINV* in *Taraxacum* flowers, representing a higher copy number of *CWIN* than that reported in *Populus*, *Arabidopsis*, *Manihot*, or *Oryza* [30,94,95,96]. The duplication of *CWIN* genes may indicate the necessity for dual sugar efflux via both merocrine and eccrine secretion models in *Taraxacum* flowers [20,48]. Unlike the two *tarSWEET9* paralogs, *tarCWIN4* and *tarCWIN6* exhibited elevated expression levels at the bud-opening stage and remained active throughout all flowering stages. During flower pollination, a sugar efflux from the epidermis suggests the preferential secretion of nectar from cells with higher cytoplasmic and apoplastic sugar concentrations, which are regulated by *tarSWEET9* in *Taraxacum* flowers [18,97]. Expression analysis of the *SPSA2* gene, which has at least two copies in flowers, showed a twofold increase in expression throughout *Taraxacum* flowering days, emphasizing its importance in sucrose biosynthesis. The significant expression of *SPSA2* enzyme levels suggests that cytoplasmic sucrose secretion is actively involved in nectar production, like findings in *Arabidopsis* [78]. Differential gene expression analysis further revealed that *tarCWIN1, tarCWIN3, tarCWIN7*, and *tarCWIN8* were up-regulated in *T. coreanum*, suggesting that *Taraxacum* species other than *T. officinale* also produce nectar during flower development. The presence of multiple *tarCWIN* gene copies in *Taraxacum* highlights their potential novel functions, arising from gene duplication [30,98].

Additionally, we were interested in identifying *MS* genes that are essential for anther and pollen development, general plant reproduction, and lipid and carbohydrate metabolism, as well as overall flower function in maize [99,100,101]. *MS* genes hold particular significance in genetic studies related to hybrid seed production, breeding for vigor, and stress adaptation [102,103,104]. Studies in *Arabidopsis* and *Oryza* have revealed their involvement in molecular pathways regulating anther and pollen development [105]. Notably, we identified two *MS* genes: *tarMS*1, which was up-regulated and expressed across all *Taraxacum* species, and *tarMS2*, which was detected only in *T. coreanum* and *T. mongolicum*, suggesting an important role for *tarMS* genes in flowering and pollination. The species-specific expression of *tarMS2* suggests that these species may rely more on pollen, rather than nectar secretion, to attract pollinators. Unlike *T. officinale*, which is widely distributed, *T. coreanum* and *T. mongolicum* are geographically restricted to East Asia, which may indicate an evolutionary adaptation involving nectar- and pollen-based specialization.

The widespread success of dandelions in harsh environments highlights the evolutionary significance of their flower development and pollination mechanisms in enabling resilience and ecological adaptability. This study discovered and predicted the nectar secretion pathway, *SWEET,* and *CWIN* gene families using comparative transcriptomic analysis in five *Taraxacum* flowers. Our results showed that the 17 *SWEET* gene families were identified in all. Eighteen *INVERTASE* genes were also identified; twelve belonged to *CWIN* and six belonged to the *vINV* class. Our study revealed that the number of *SWEET* genes in *Taraxacum* is less than that reported for other Angiosperm species, but at the whole genomic DNA level. However, *CWIN* genes were highly duplicated and expressed in *Taraxacum* flowers, indicating that the nectar secretion pathway results in greater sugar solution efflux through eccrine routes in *Taraxacum*. Finally, we suggest that identifying *tarMS* genes in *Taraxacum* (*T. coreanum* and *T. monogynam*) might provide a piece of information to study the two independent pollination systems in *Taraxacum* species. Our findings lay the groundwork for further research into the function and contribution of *SWEET* and *CWIN* genes in plant nectar secretion systems and their role during flower development in *Taraxacum* species.

## 4. Materials and Methods

### 4.1. Plant Materials, RNA Sequencing, and Assembly

Five species of the *Taraxacum* genus—*T. coreanum*, *T. officinale*, *T. mongolicum*, *T. hallaisanense*, and *T. ohwianum*—were selected for this study, as they are naturally distributed in South Korea (plant collection locations listed in Supplementary Table S1). Flowers at 12 h post-anthesis were harvested for RNA extraction using the single-step RNA isolation method [106]. The extracted total RNA was used for cDNA library construction with the TruSeq Stranded mRNA kit. High-throughput sequencing was performed using the Illumina HiSeq 2500 platform at the Phyzen Genomics Institute (South Korea), generating paired-end reads. Quality control of the raw reads was assessed using FastQC v0.11.9, and adapter trimming was performed using Trimmomatic v0.40 [107]. A combined dataset of paired-end reads from the five *Taraxacum* species was assembled de novo using Trinity v2.12.0 [108]. Super-Transcripts, representing unigenes with exon structures while minimizing redundancy, were generated using the Trinity v2.12.0 pipeline [108,109]. The quality of assembled transcripts was evaluated using BUSCO v5.1.2 with the eudicot lineage dataset [110]. The overall methodological workflow of this study is outlined in Supplementary Figure S1.

### 4.2. Gene Annotation

TransDecoder v5.5.0 (https://github.com/TransDecoder/TransDecoder, 4 February 2022) was used to predict candidate protein-coding regions from the assembled transcripts. Reference sequences for sucrose biosynthesis, pollen development, and nectar synthesis (58 genes) were obtained from the TAIR database [111] (Supplementary Table S2). Additionally, 59 genes related to sugar biosynthesis, including *SWEET* (*SWT*) genes, were retrieved from the TAIR and Swiss-Prot databases [112]. BLASTX and BLASTP searches were conducted for functional annotation. KEGG pathway analysis was performed using the longest open reading frames (ORFs) predicted by TransDecoder to categorize unigenes into metabolic pathways, including sucrose biosynthesis [113,114,115]. Candidate *Taraxacum* cell wall invertase (*CWIN*) unigenes were identified through BLAST searches against *Arabidopsis thaliana CWIN4*. Conserved *CWIN* motifs, active sites, and glycosylation sites were analyzed using MUSCLE alignment in Geneious R11, and a phylogenetic tree was constructed using IQ-TREE v2.2.0 with 10,000 bootstrap replicates [116,117]. Phylogenetic visualization was performed using FigTree v1.4.4 (http://github.com/rambaut/figtree/, accessed on 2 April 2022).

### 4.3. Differential Gene Expression Analysis

Differential gene expression analysis was conducted using RSEM (RNA-Seq by Expectation Maximization), an accurate quantitative method for estimating transcript abundances [118]. A combined *Taraxacum* transcriptome was assembled in a single run using the Trinity assembler [108]. Differential expression analysis was performed using a contrast-based approach, where *T. officinale* served as the reference species for the comparative expression analysis of nectar synthesis-related genes across other *Taraxacum* species. The EdgeR package was used to identify differentially expressed genes (DEGs) with a false discovery rate (FDR) cut-off of 0.05, log-fold changes (>2 and >10FC), and a dispersion parameter of 0.1 [119]. Visualization of MA and volcano plots was conducted in R Studio (2022.05.08). Additionally, the NOISeq pipeline (https://www.bioconductor.org/packages/release/bioc/html/NOISeq.html, accessed on 18 February 2022) was employed to assess overall DEG patterns using a non-parametric approach for up- and down-regulated gene comparisons among *Taraxacum* species [120].

### 4.4. Quantitative PCR Expression Studies

To validate the expression patterns of *SWEET*, *CWIN*, and other nectar-related genes, candidate genes were first identified via BLAST searches against the TAIR database [111,114,115]. Complete ORFs and conserved domains (CDs) were confirmed using Geneious R11 (https://www.geneious.com/prime/, accessed on 19 May 2022). The *tarSWEET9*, *tarCWIN4*, *tarSPAS*, and *tarSUS* genes were selected for primer design, and primer specificity was evaluated using Primer3 (default parameters, melting temperature of 60 °C) in Geneious R11. Quantitative real-time PCR (qRT-PCR) was performed to examine gene expression across different flower development stages (days 1–6), as well as at specific time points: closed buds (3:00 AM) and buds opened (8:00 AM). Total RNA was extracted from *T. officinale* flowers using the RNeasy Plant Mini Kit (Qiagen Inc., Germany), and cDNA was synthesized using the TOPscript™ cDNA Synthesis Kit (Enzynomics, Daejeon, South Korea). Reverse transcription was conducted in a 10 µL reaction at 70 °C for 5 min using an Oligo (dT) 15 primer. qPCR was performed using the GoTaq^®^ qPCR Master Mix (A6001, Promega) under standard cycling conditions on an Applied Biosystems 7500 Step OnePlus system, with triplicate technical replicates. Actin and elongation factor genes were selected as reference controls based on the complete ORF structure, and primers were designed for normalization analysis (Table S7). Leaf and root tissues of *T. officinale* were used as comparative controls, with water serving as the negative control. DEGs, including *tarSWEET9* and *tarCWIN4*, were analyzed for expression profiles at different time points to validate their roles in the nectar secretion pathway. Quantitative real-time PCR (qPCR) data were analyzed using one-way analysis of variance (ANOVA) to assess differences in gene expression across floral developmental stages. When the ANOVA indicated significant variation (*p* < 0.05), Tukey’s Honest Significant Difference (HSD) post hoc test was performed to determine pairwise differences between groups [121]. The significance of gene expression was calculated using a *t*-test in R (stats package, version 3.6.2, accessed on 30 April 2022).

## 5. Conclusions

This study provides the first comprehensive transcriptomic and expression-based analysis of nectar secretion-related genes in five *Taraxacum* species, focusing on the key roles of *SWEET* and *CWIN* gene families. Among the 17 *SWEET* and 18 *CWIN* genes identified, *SWEET*9 and *CWIN*4 were consistently up-regulated during flowering, indicating their central function in nectar secretion across species. qPCR validation in *T*. *officinale* further confirmed that *SWEET*9, *CWIN*4, CWIN6, and *SPAS*2 exhibit significant expression during floral development, with marked down-regulation after pollination, suggesting a pollination-responsive regulation mechanism. The relatively high expression of these genes in *T*. *officinale* correlates with its dual provision of nectar and pollen and its successful global distribution. In contrast, reduced gene expression after pollination reflects a potential resource-conservation strategy once fertilization is achieved. Together, these results highlight how nectar secretion is finely tuned by genetic regulation and how such regulation may influence pollinator interactions and species divergence. By integrating transcriptomic analysis with gene expression profiling, this study offers new insights into the molecular basis of nectar production and lays a foundation for future research on the evolution of floral traits and pollination strategies in *Taraxacum* and other members of the Asteraceae family.

## Figures and Tables

**Table 1:** Assembly and gene annotation statistics of *Taraxacum* unigenes.

S.no	Species	Longest Transcripts	N50	Unigenes	Cd-Hit-Est Unigenes	TAIR	Swiss-Prot	KEGG
1	*T. ohwianum*	122,494	1635	64,296	27,691	16,431	47,659	35,261
2	*T. officinale*	120,911	938.75	58,924	29,511	15,782	35,788	35,165
3	*T. mongolicum*	129,433	1053.75	69,234	32,706	22,900	48,092	39,386
4	*T. coreanum*	156,377	929.84	73,698	31,318	24,678	69,375	44,722
5	*T. hallaisanense*	96,360	927.65	59,599	29,039	17,897	33,334	28,375
6	combined	397,344	1304	197,473	58,339	53,564	65,149	58,783

## Data Availability

The datasets supporting the results of this article are included in the additional files. Unique-Transcripts sequences are available in GenBank (ON351066-ON351241), and whole transcriptome sequencing data are available in the NCBI-SRA database (SRR32995710-14).

## Supplementary Materials

The following supporting information can be downloaded at: https://www.mdpi.com/article/10.3390/plants14111718/s1, Supplementary Figure S1. Overview of the RNA-Seq analysis pipeline used in this study. Supplementary Figure S2. BUSCO assessment and summary of *Taraxacum* transcriptome unigene data. Supplementary Figure S3. Gene Ontology (GO) analysis of sugar metabolism-related terms in *Taraxacum* using TAIR database BLAST analysis: (A) biological process, (B) molecular function, and (C) cellular components. Supplementary Figure S4. Kyoto Encyclopedia of Genes and Genomes (KEGG) pathway analysis of all *Taraxacum* unigenes. (B) Nectar compound-related unigenes in the KEGG pathway. Supplementary Figure S5. (A) Differentially expressed gene (DEG) analysis of unigene expression in the *Taraxacum* genus using NOISeq. (B) Differentially expressed genes in different *Taraxacum* species compared to *T*. *officinale*. tha: *T. hallaisanense*; tmo: *T. mongolicum*; toh: *T. ohwianum*; tof: *T*. *officinale*; tco: *T. coreanum*. Supplementary Figure S6. Heatmap of differential expressions (log10 fold change) in *Taraxacum* flowers. Supplementary Table S1. List of *Taraxacum* species, including species collection details and specimen numbers. Supplementary Table S2. TAIR database BLAST results for nectar secretion and sucrose metabolism-related unigenes in *Taraxacum* flowers. Supplementary Table S3. Differentially expressed *tarCWIN* unigenes in *Taraxacum* flowers analyzed using edgeR (log2 fold change). Supplementary Table S4. DEGs of tarCWIN unigenes compared to all *Taraxacum* flowers using the edgeR tool at log2 fold change. Supplementary Table S5. Differentially expressed *tarMS* unigenes in *Taraxacum* flowers analyzed using edgeR (log2 fold change). Supplementary Table S6. Differential expression analysis of all nectar secretion pathway unigenes in *Taraxacum* flowers using edgeR (log2 fold change). Supplementary Table S7. The primer list used for unigene analysis in the nectar secretion pathway.
