# Supplementary material for: Unraveling the Nectar Secretion Pathway and Floral-Specific Expression of SWEET and CWIV Genes in Five Dandelion Species Through RNA Sequencing
Source: Plants (Basel). 2025 Jun 5;14(11):1718. doi: 10.3390/plants14111718 (PMC12156968; doi:10.3390/plants14111718)
Supplement: Supplementary file 1 [file plants-14-01718-s001.zip › Supplementary_Material.pdf]

Supplementary Material

Supplementary Data

Supplementary Figure S1. Overview of the RNA-Seq analysis pipeline used in this study.

Supplementary Figure S2. BUSCO assessment and summary of *Taraxacum* transcriptome unigene data.

Supplemeantay Figure S3. ene Ontology (GO) analysis of sugar metabolism-related terms in *Taraxacum* using TAIR database BLAST analysis.
(S3A) Biological Process, (S3B) Molecular Function, and (C) Cellular Components.

Supplementary Figure S4. S4A-Kyoto Encyclopedia of Genes and Genomes (KEGG) pathway analysis of all *Taraxacum* unigenes.
(S3B) Nectar compound-related unigenes in the KEGG pathway.

Supplementary Figure S5. Differentially expressed gene (DEG) analysis of unigene expression in the *Taraxacum* genus using NOISeq.
(S**5B**) Differentially expressed genes in different *Taraxacum* species compared to *T. officinale.*

tha: *T. hallaisanense*, **tmo**: *T. mongolicum*, **toh**: *T. ohwianum*, **tof**: *T. officinale*, **tco**: *T. coreanum.*

Supplementary Figure S6. Heatmap of differential expression (log10-fold change) in *Taraxacum* flowers.

Supplementary Table S1. List of *Taraxacum* species, including species collection details and specimen numbers.

Supplementary Table S2. TAIR database BLAST results for nectar secretion and sucrose metabolism-related unigenes in *Taraxacum* flowers.

Supplementary Table S3. Differentially expressed *tarCWIN* unigenes in *Taraxacum* flowers analyzed using edgeR (log2 fold change).

Supplementary Table S4. DEGs of *tarCWIN* unigenes compared to all *Taraxacum* flower using edgeR tool at log2fold change.

Supplementary Table S5. Differentially expressed *tarMS* unigenes in *Taraxacum* flowers analyzed using edgeR (log2 fold change).

Supplementary Table S6. Differential expression analysis of all nectar secretion pathway unigenes in *Taraxacum* flowers using edgeR (log2 fold change).

Supplementary Table S7. Primer list used for unigene analysis in the nectar secretion pathway.

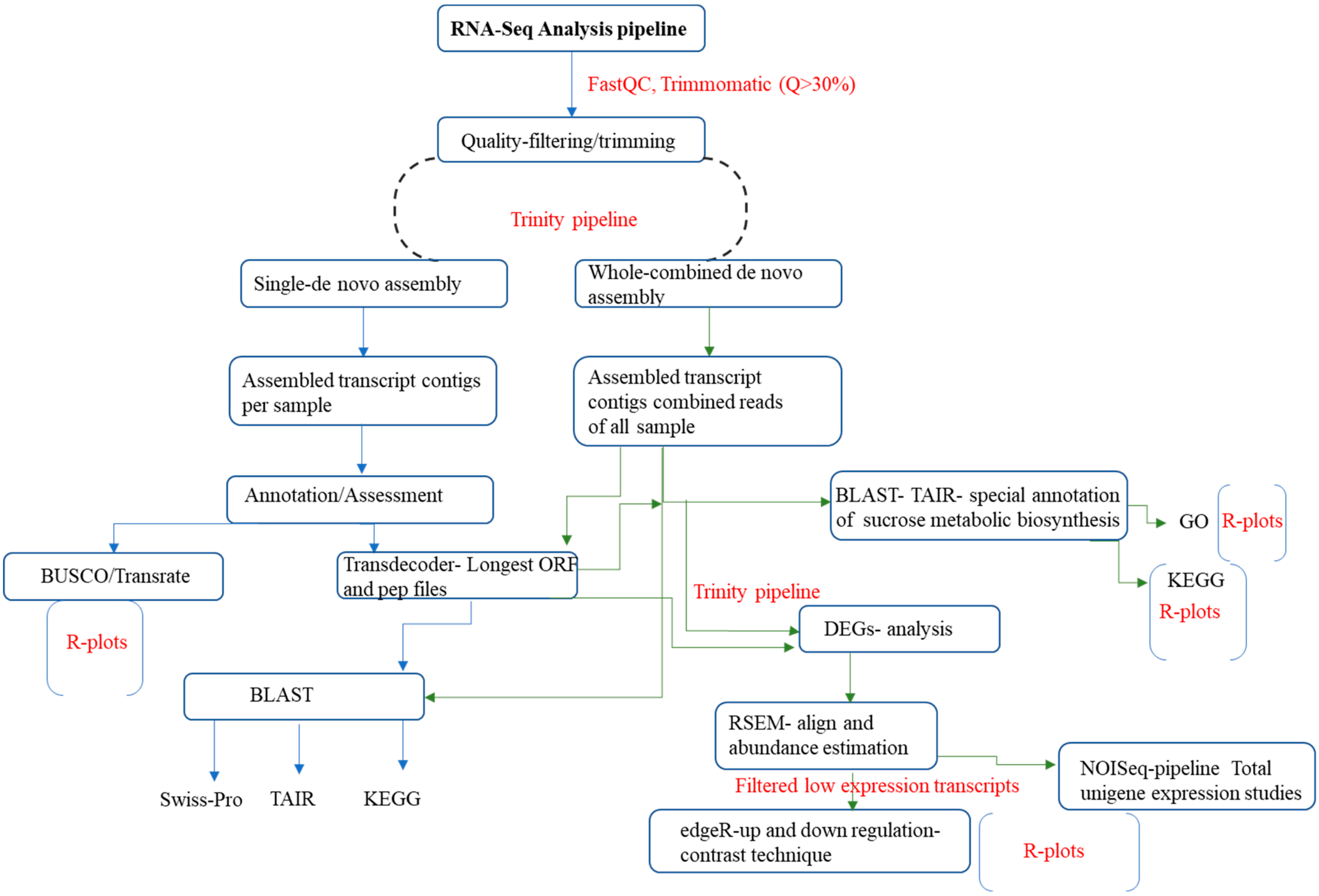

Supplementary Figure S1. The outline of the RNA-Seq Analysis pipeline used in this study.

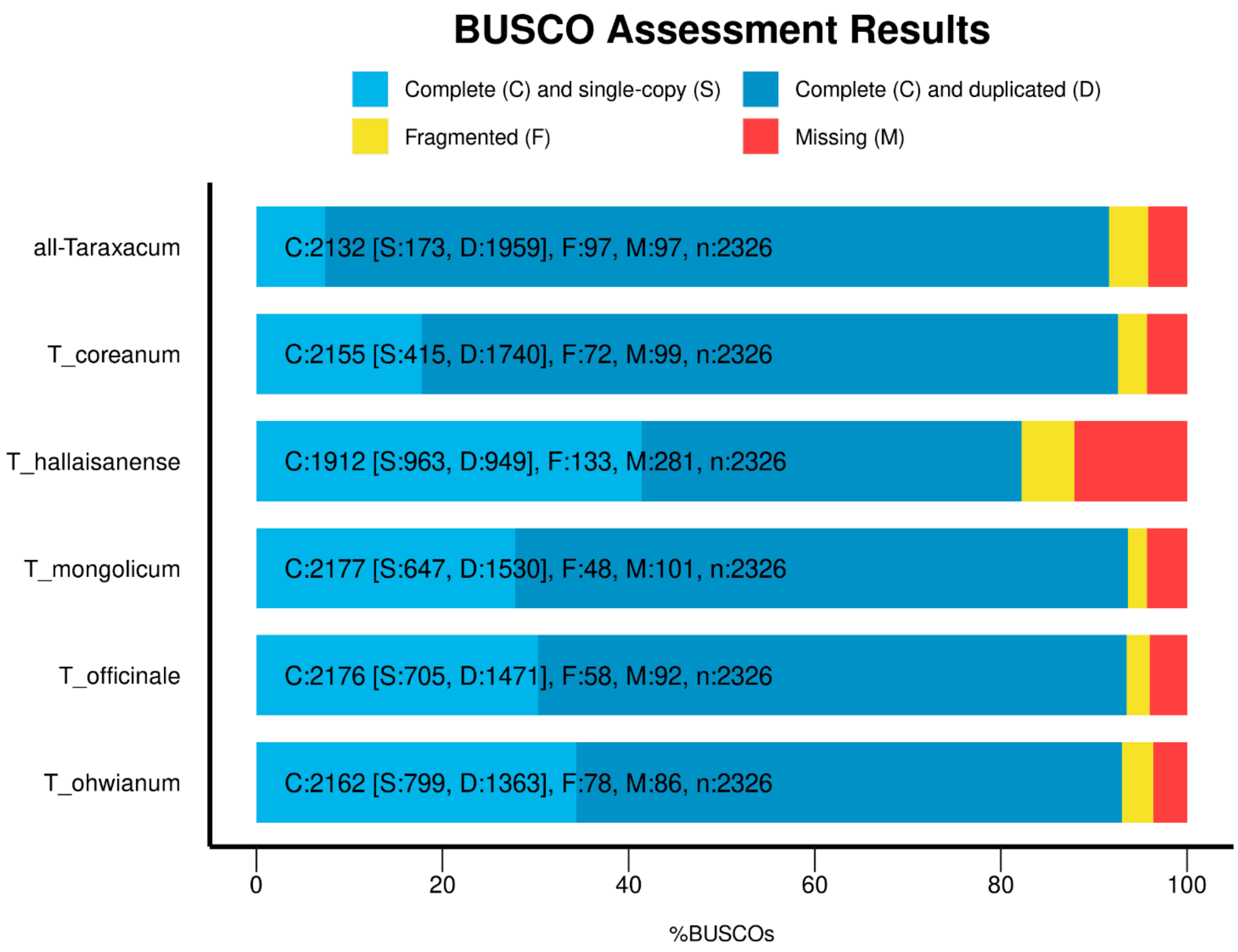

Supplementary Figure S2. BUSCO assessment and summaries for *Taraxacum* transcriptome unigenes data.

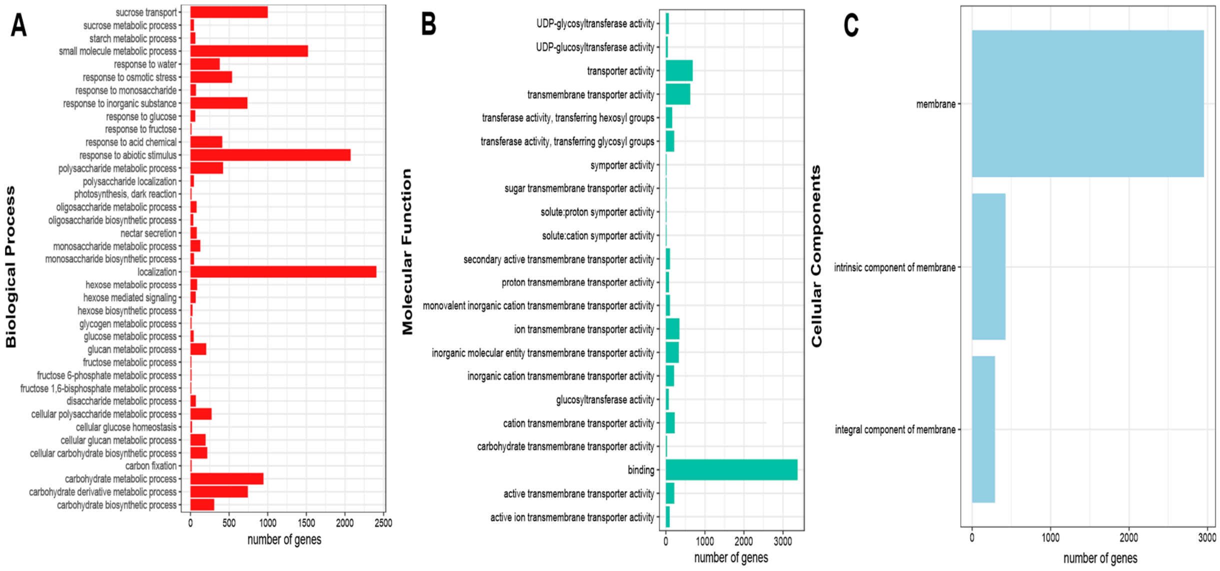

Supplementary Figure S3. Geno ontology (GO)- sugar metabolism terms of *Taraxacum* using TAIR database blast analysis. S3A. Biologial process, S3B. Molecular Function and C. Cellular Components.

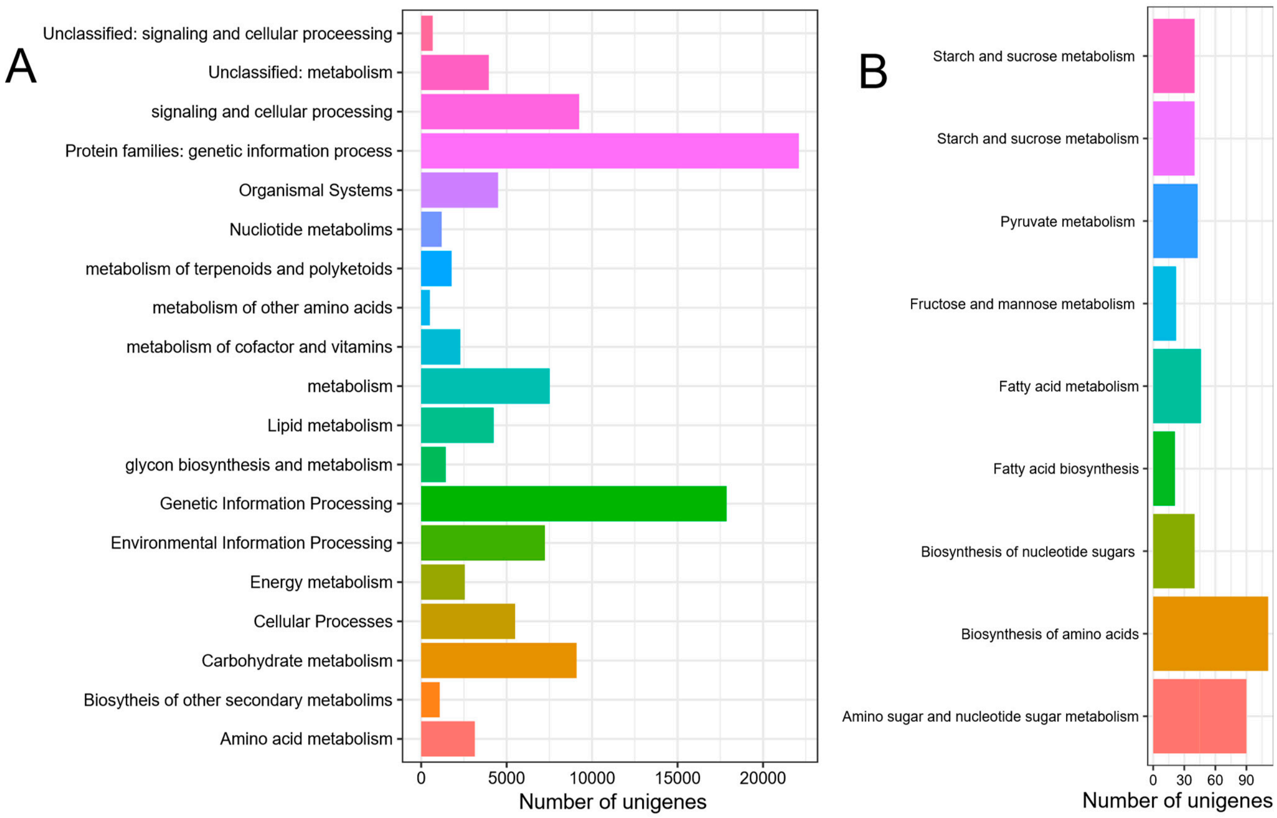

Supplementary Figure S4A. Kyoto Encyclopedia of Genes and Genomes (KEGG) pathway plots of all unigenes of *Taraxacum*. S4B. Nectar compound-related unigenes in the KEGG pathway.

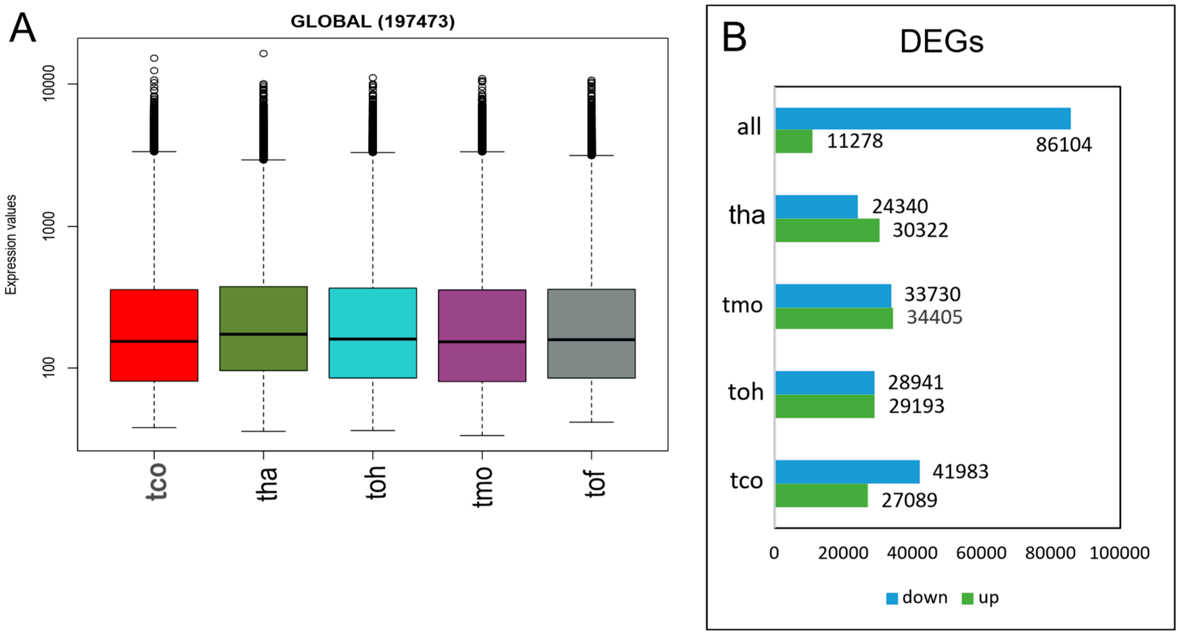

Supplementary Figure 5A. DEGs analysis using NOISeq of unigene expressions in *Taraxacum* genus. **5B.** Differentially expressed genes *Taraxacum* genera species versus *T. officinale*. tha: *T. hallaisanense*, tmo: *T. mongolicum*, toh: *T. ohwianum* , tof: *T. officinale* and tco: *T. coreanu*

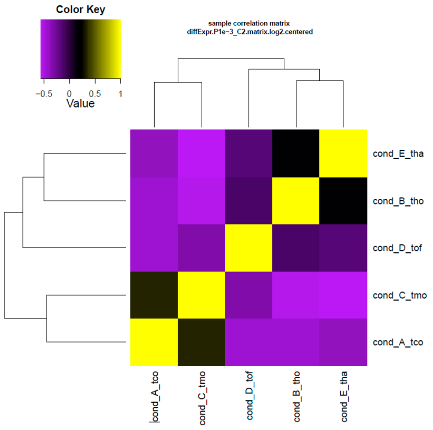

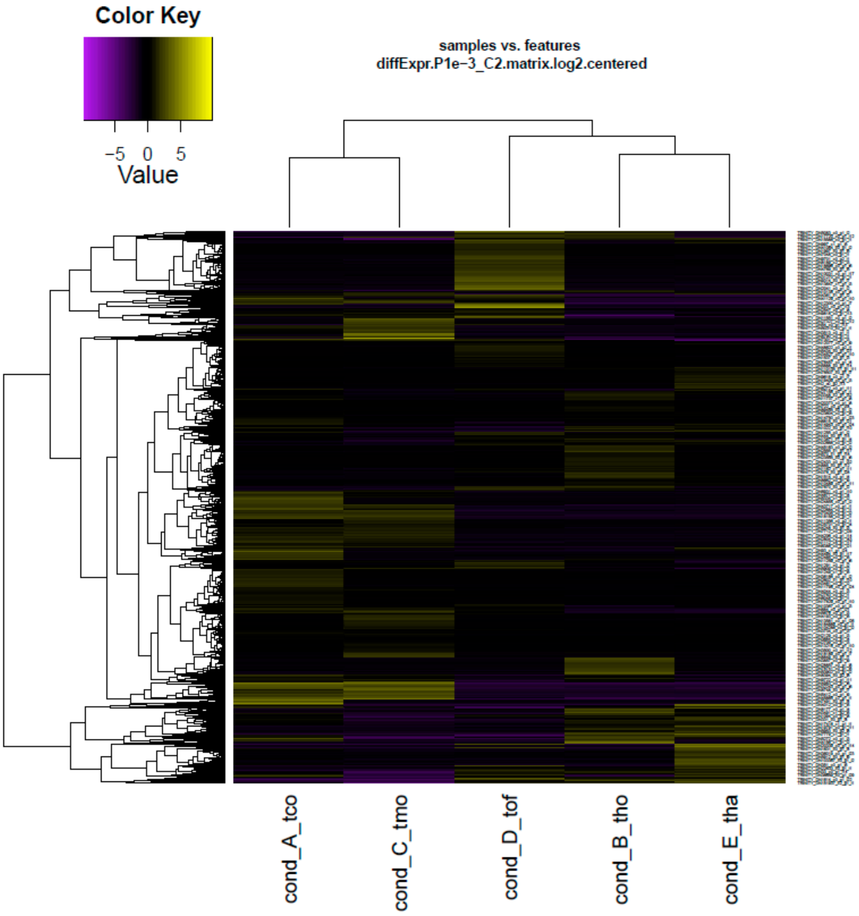

Supplementary Figure 6. Differential expression comparison (log10-fold change) heat map plots in *Taraxacum* flowers.
